# Ritlecitinib, a JAK3/TEC family kinase inhibitor, stabilizes active lesions and repigments stable lesions in vitiligo

**DOI:** 10.1007/s00403-024-03182-y

**Published:** 2024-07-18

**Authors:** Yuji Yamaguchi, Elena Peeva, Ester Del Duca, Paola Facheris, Jonathan Bar, Ronald Shore, Lori Ann Cox, Abigail Sloan, Diamant Thaçi, Anand Ganesan, George Han, Khaled Ezzedine, Zhan Ye, Emma Guttman-Yassky

**Affiliations:** 1 Inflammation & Immunology Research Unit, Pfizer, Collegeville, PA USA; 2 Inflammation & Immunology Research Unit, Pfizer, Cambridge, MA USA; 3 Department of Dermatology, and Laboratory of Inflammatory Skin Diseases, Icahn School of Medicine, Mount Sinai, New York, NY USA; 4 IRCCS Humanitas Research Hospital, Milan, Italy; 5 Faculty of Medicine, Tel-Aviv University, Tel-Aviv, Israel; 6 Ronald N. Shore Dermatology, Rockville, MD USA; 7 Clinical Statistics, Pfizer, Cambridge, MA USA; 8 Institut fuer Entzuendungsmedizin, University of Luebeck, Luebeck, Germany; 9 Department of Dermatology, University of California, Irvine, Irvine, CA USA; 10 Department of Dermatology, Zucker School of Medicine at Hofstra/Northwell, New Hyde Park, NY USA; 11 Department of Dermatology, Hôpital Henri Mondor, Créteil, France

**Keywords:** Ritlecitinib, Vitiligo, Non-segmental vitiligo, JAK inhibitor, Biomarkers

## Abstract

The efficacy of ritlecitinib, an oral JAK3/TEC family kinase inhibitor, on active and stable lesions was evaluated in patients with active non-segmental vitiligo in a phase 2b trial (NCT03715829). Patients were randomized to placebo or daily ritlecitinib 50 mg (with or without 4-week 100-mg or 200-mg loading dose), 30 mg, or 10 mg for 24 weeks. Active lesions showed greater baseline expression of inflammatory/immune markers *IFNG* and *CCL5*, levels of CD103, and T-cell infiltrates than stable lesions. Patients with more active than stable vitiligo lesions showed higher baseline serum levels of CXCL9 and PD-L1, while patients with more stable than active lesions showed higher baseline serum levels of HO-1. At Week 24, ritlecitinib 50 mg significantly stabilized mean percent change from baseline in depigmentation extent in both active lesions and stable lesions vs. placebo-response, with stable lesions showing greater repigmentation. After 24 weeks of treatment, ritlecitinib 50 mg increased expression of melanocyte markers in stable lesions, while Th1/Th2-related and co-stimulatory molecules decreased significantly in both stable and active lesions. Serum from patients with more active than stable lesions showed decreased levels of ICOS and NK cell activation markers. These data, confirmed at transcription/protein levels, indicate that stable lesion repigmentation occurs early with ritlecitinib, while active lesions require stabilization of inflammation first. ClinicalTrials.gov: NCT03715829.

**Supplementary Information:**

The online version contains supplementary material available at 10.1007/s00403-024-03182-y.

## Introduction

Vitiligo is an autoimmune disease characterized by depigmentation in the skin, hair, or both, with a global burden affecting an estimated 0.5-2% of the population [1, 2]. In patients with vitiligo, CD8^+^ T cells attack melanocytes, resulting in non-scaly chalky-white amelanotic lesions [3, 4].

An abnormal adaptive immune response from cytotoxic CD8^+^ T cells produces cytokines typical of T helper Types 1, 2, and 17 (Th1, Th2, and Th17) immune responses, including interferon (IFN)-γ [5, 6]. The Th1 and Th2 immune responses in turn activate the Janus kinase/signal transducers and activators of transcription (JAK/STAT) pathway [7], leading to increased levels of IFN-γ that drives decreased melanocyte adhesion [8]. Furthermore, chemokines such as C-X-C motif chemokine ligand (CXCL) 9, CXCL10, and CXCL11 are released, creating a positive feedback loop [5, 9, 10].

Patients with non-segmental vitiligo (NSV) may have stable lesions, which may remain unchanged for at least 6 months, and active lesions that continue to progress simultaneously depending on disease activity [11]. Previous publications have described differences between patients with stable lesions and patients with active lesions at the serological and biochemical levels [12]. Skin biopsy studies have also shown differences, including: (1) greater vacuolar changes of basal keratinocytes and lymphocyte infiltration in the upper dermis, (2) lower adhesion of melanocytes to collagen type IV, and (3) higher expression levels of caspase 3, annexin V, superoxide dismutase, glutathione peroxidase, and malondialdehyde in patients with active lesions vs. patients with stable lesions [12]. In several studies over the past decade, patients with active lesions (compared with patients with stable lesions) show: decreased blood regulatory T cells (Tregs) and CD4^+^/CD8^+^ T-cell ratio [13], increased serum CXCL9 and CXCL10 [14], increased serum CCL20 [15], increased CD8^+^ T cells and expression of E-cadherin in the epidermis and dermis [16], increased CXCL10 in peri-lesional skin [17], and increased CXCL9 and CXCL10 in suction blister fluid [18]. These findings may indicate that active and stable lesions display differential profiles of immune cells and activation of the JAK/STAT pathway.

Current treatments for vitiligo often have limited efficacy and/or undesirable side effects [19–23]. Interest in JAK/STAT pathway inhibition downstream of disease-associated cytokines such as IFN-γ as a potential treatment for vitiligo has grown in recent years and resulted in several clinical trials [24–28] and the approval of ruxolitinib cream, a topical JAK1/2 inhibitor for the treatment of NSV [28, 29].

Ritlecitinib, an oral selective inhibitor of JAK3 and the tyrosine kinase expressed in hepatocellular carcinoma (TEC) family kinases [25], is approved for the treatment of alopecia areata [30], and is currently under investigation for the treatment of NSV. In a phase 2b trial (NCT03715829), ritlecitinib demonstrated significant improvement in the Facial Vitiligo Area Scoring Index and immune and melanocyte biomarkers at Week 24 in patients with active NSV [31, 32]. The main objectives of this report were to evaluate the efficacy of ritlecitinib vs. placebo on active and stable lesions in patients with vitiligo who participated in the phase 2b trial, and to explore the effect of ritlecitinib on melanocyte and immune biomarkers in active and stable lesions.

## Methods

### Trial design and patients

This was an exploratory analysis of the randomized, double-blind, placebo-controlled, 24-week dose-ranging period of the phase 2b NCT03715829 study [31]. The study protocol and all other documentation were approved by each study center’s institutional review board or independent ethics committee. This study followed the Declaration of Helsinki and the good clinical practice guidelines put forth by the International Conference on Harmonization. All patients provided written informed consent.

Inclusion criteria included adult patients with active NSV who had ≥ 1 active vitiligo lesion. Other criteria included a body surface area (BSA) of 4–50% (excluding acral lesions) and facial BSA > 0.25% (excluding vermilions) at screening and baseline visits.

Cohort 1: In the dose-ranging period of the study, patients were randomized to receive daily ritlecitinib for 24 weeks, with or without a 4-week loading dose: 200 mg (loading dose)/50 mg, 100/50 mg, 50 mg, 30 mg, 10 mg, or placebo.

Cohort 2: Skin biopsies were taken at baseline and at Week 24 and analyzed by quantitative real-time PCR (qPCR), TaqMan Low Density Array (TLDA), RNA-seq, and immunohistochemistry (IHC) for melanocyte, Th1/Th2 markers, and co-stimulatory molecules as previously described [32]. Patients with a greater number of active lesions than stable lesions were considered “patients with more active than stable lesions”, while patients with a greater number of stable lesions were considered “patients with more stable than active lesions”. Patients with similar numbers of both active and stable lesions were excluded from these analyses.

### Outcomes

Central photograph reviewers categorized all lesions as either active or stable, then evaluated depigmentation extent (100%, 90%, 75%, 50%, 25%, or 10%) at baseline and at Week 24 using photographs of both active lesions and stable lesions other than on the face as target lesions. Then each mean percent change from baseline (%CFB) was measured.

#### qPCR

TLDA cards (Thermo Fisher) were used for qPCR. Eukaryotic 18 S recombinant RNA was used as an endogenous control. Expression values were normalized to *Rplp0.*

#### IHC

IHC was performed on frozen skin sections as previously described [33, 34], using purified mouse anti-human antibodies (Table S1).

### Serum protein quantification

Blood was collected, centrifuged, and stored at − 80^o^C. Aliquots were analyzed by the ultrasensitive proteomic OLINK Proseek multiplex assay (a proximity extension assay using oligonucleotide-labeled antibody probe pairs), using target cardiovascular disease II, cardiovascular disease III, immuno-oncology, and neurology multiplex panels, as previously described [35, 36].

### Lesion sample status evaluation

Central photograph reviewers determined the overall activity status of all lesions at baseline and at all timepoints. Active lesions were defined as progressive and one of the following: confetti-like lesions, trichrome lesions, and Koebner phenomenon types 2a/b [37], while stable lesions were identified as lesions that did not show active signs (absence of confetti-like pattern, trichrome appearance, and Koebner phenomenon type 2a/2b).

### Statistical analysis

The least squares mean with 90% CI was calculated for depigmentation using an analysis of covariance model at Week 24, which included treatment, baseline central read rate, and Fitzpatrick skin type as covariates. For all analyses here, the 200/50-mg, 100/50-mg, and 50-mg groups were pooled. One-sided unadjusted *P*-values were reported.

RNA-seq data were log-2 transformed with voom transform [38] and modeled using a mixed-effect model with time, treatment, and tissue interaction as a fixed effect and a random effect for each participant (using the R LIMMA package). The Benjamini-Hochberg procedure was used to adjust *P*-values for multiple hypotheses by controlling the FDR. FDR < 0.1 was determined as a criterion for overall statistically significant findings. Normalized PCR values and serum protein measures (normalized protein expression) were also modeled using a mixed-effect model with time, treatment, and tissue interaction as a fixed effect and a random effect for each participant (using the R nlme [nonlinear mixed-effects] package). Gene set variation analysis (GSVA; z-score) was also used to evaluate different immune pathways. For baseline serum protein analyses, a linear model was utilized to evaluate differential proteins between stable and active lesions. A linear mixed model was used to evaluate changes in protein levels from baseline by treatment group, timepoint, and lesion type.

## Results

### Baseline characteristics

Cohort 1: in total, 364 patients were randomized to daily ritlecitinib 50 mg (with or without a 4-week 100-mg or 200-mg daily loading dose; *N* = 199), 30 mg (*N* = 50), 10 mg (*N* = 49), or placebo (*N* = 66) (Table S2; [31]). A total of 298 patients completed the dose-ranging period.

Cohort 2: 65 patients participated in a skin biopsy sub-study [32]. Patients in the sub-study were on average 44.9 years old, 52% were female, and 68% were White (Table S3). Mean (SD) disease duration was 19.8 (12.5) years. Out of 65 patients, 31 patients had more active than stable lesions, and 27 patients had more stable than active lesions. Seven patients had similar numbers of active and stable lesions and were excluded from the analyses.

### Baseline biomarkers comparisons

To examine the differences between active lesions and stable lesions at baseline, each sample was categorized as active or stable lesion based on photograph review, followed by RNA-seq, qPCR, and IHC. 136, 33, 32, and 56 pairs of target lesions were evaluated from the 50-mg, 30-mg, 10-mg, and placebo groups, respectively. 183, 28, 44, and 62 pairs of target lesions (at baseline and at Week 24) were evaluated from the 50-mg, 30-mg, 10-mg, and placebo groups, respectively.

**Fig. 1:**
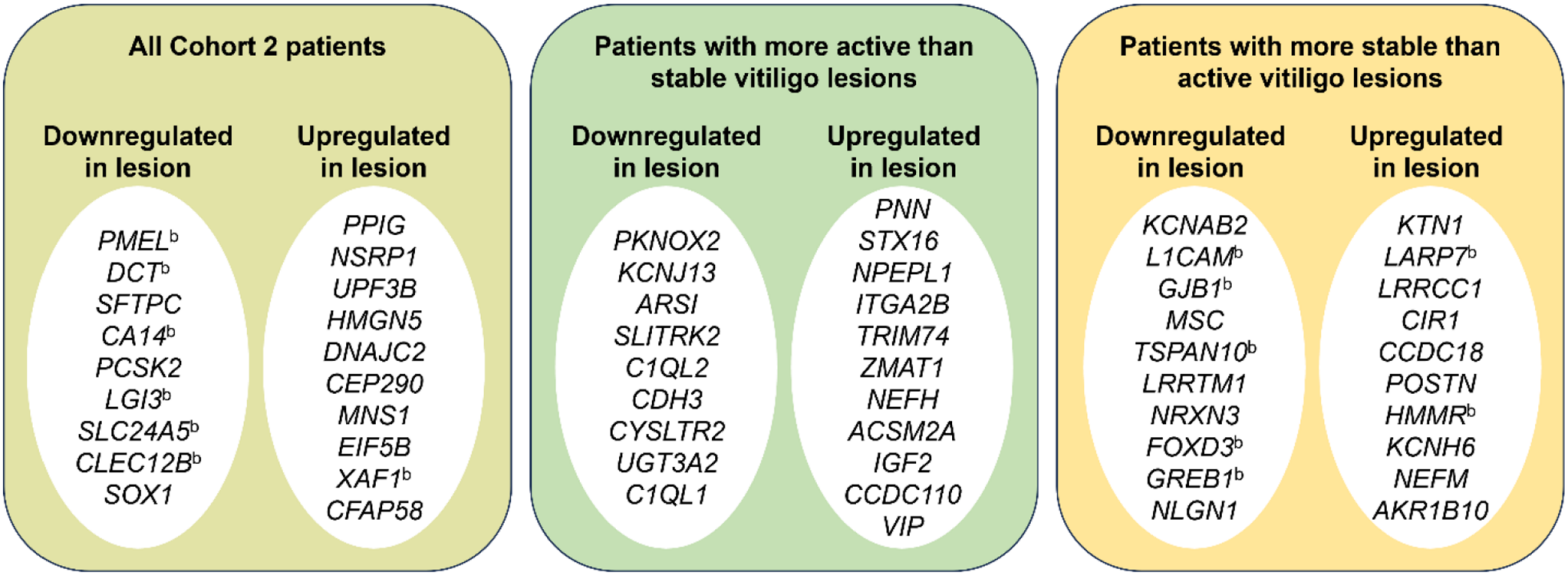
Top 10 genes^a^ upregulated or downregulated in vitiligo lesions as compared with non-lesional skin at baseline from all patients in Cohort 2, patients with more active than stable lesions, and patients with more stable than active lesions. ^a^ Defined as *FDR* < 0.05. ^b^ Gene associated with melanocyte development/regulation or vitiligo (based on PubMed search)

When examining biopsied skin samples from Cohort 2 by RNA-seq at baseline, no statistically significant genes were found when comparing: (1) between lesions from patients with more active than stable lesions and lesions from patients with more stable than active lesions (Fig. S1a), (2) between non-lesional skin from patients with more active than stable lesions and non-lesional skin from patients with more stable than active lesions (Fig. S1a), or (3) between “lesions vs. non-lesions in patients with more active than stable lesions” vs. “lesions vs. non-lesions in patients with more stable than active lesions” (“delta-delta” comparisons; Fig. S1b). Statistically significant genes were observed in “delta” comparisons of differences in “lesions vs. non-lesions” in patients with more active than stable lesions, patients with more stable than active lesions, or all patients in Cohort 2. Overall, non-lesional skin displayed significantly higher expression of melanocyte function- and development-associated genes such as *PMEL, DCT*, and *SLC24A5*, while lesions showed upregulation of apoptosis-associated genes such as *XAF1* (Fig. 1a **left**, Table S4, false discovery rate [FDR] < 0.05). When comparing lesions and non-lesional skin in patients with more active than stable lesions, a different set of genes was significantly upregulated or downregulated (Fig. 1a **middle**, Table S4, FDR < 0.05). When comparing lesions and non-lesional skin in patients with more stable than active lesions, a third set of genes was significantly upregulated or downregulated. Melanocyte-associated genes such as *L1CAM, FOXD3*, and *GREB1* showed significantly increased expression in non-lesional skin (Fig. 1a **right**, Table S4, FDR < 0.05). Compared with non-lesional skin, lesional skin showed significantly increased expression of genes such as *LARP7*, and *HMMR*.

qPCR and TLDA showed that active lesions expressed higher gene levels of *IFNG* and *CCL5* than stable lesions (Fig. S2, *P* < 0.05), although this trend was not reflected by RNA-seq.

**Fig. 2:**
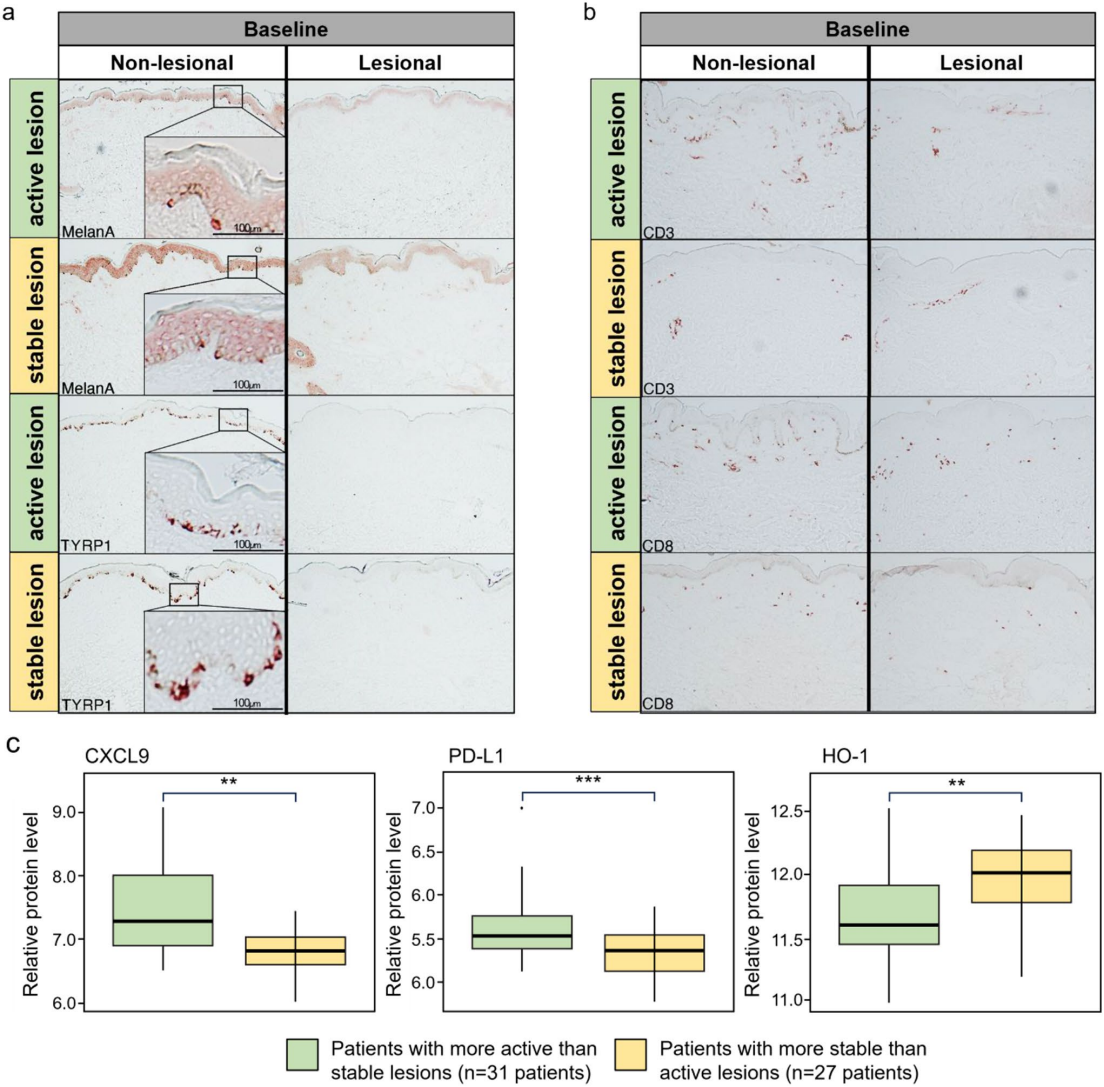
(**a**) Melanocyte marker and (**b**) CD3 and CD8 protein levels by IHC at baseline in active lesions and stable lesions in patients in Cohort 2. (**c**) Differential protein levels in blood serum of patients with more active than stable lesions and patients with more stable than active lesions (Cohort 2) as measured by proteomics at baseline. **P < 0.01, ***P < 0.001, patients with more active than stable lesions vs. patients with more stable than active lesions

At the protein level at baseline measured by IHC, stable and active lesions showed similar expression levels of melanocyte markers (Fig. 2a), but active lesions tended to contain greater numbers of CD3/CD8^+^ T-cell infiltrates than stable lesions (Fig. 2b, *P* < 0.1). The only difference noted was when measured by IHC, epidermal CD103 expression levels were higher in active lesions vs. stable lesions (Fig. S2, *P* < 0.05).

In patients with more active than stable lesions, significantly higher serum blood protein levels of CXCL9 (*P* = 0.004) and PD-L1 (*P* = 0.0007) were observed at baseline compared with patients with more stable than active lesions (Fig. 2c) as measured by proteomics, while HO-1 serum protein levels were significantly higher in patients with more stable than active lesions (*P* = 0.002).

### Efficacy comparison between active lesions and stable lesions following ritlecitinib

**Fig. 3:**
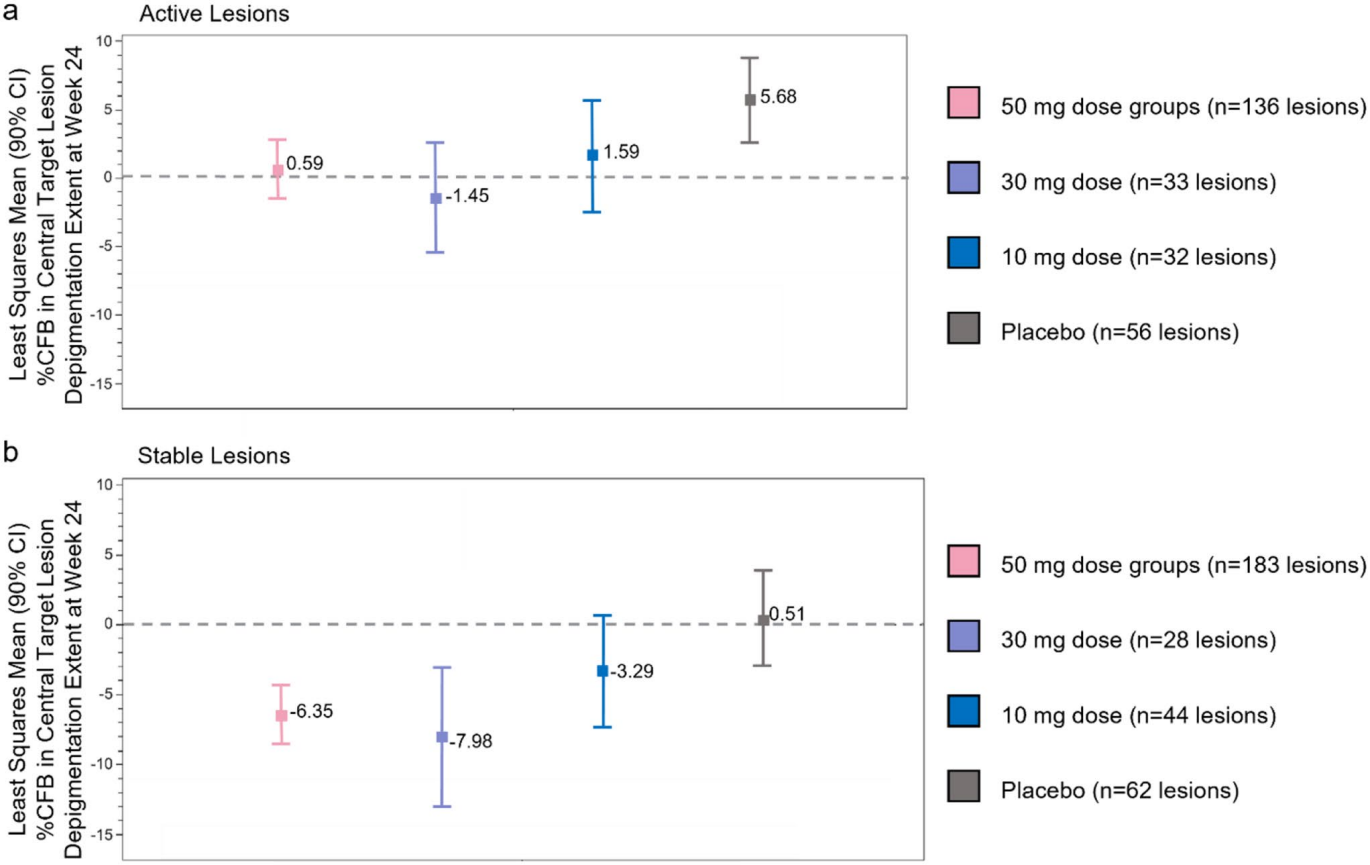
Mean %CFB of clinical depigmentation in (**a**) active lesions and (**b**) stable lesions at Week 24 in Cohort 1. %CFB, percent change from baseline; CI, confidence interval

We next investigated whether there were differences in repigmentation between active and stable lesions following 24 weeks of ritlecitinib treatment (excluding lesions on the face). For all patients enrolled in the phase 2b trial, within active lesions, ritlecitinib resulted in statistically significant reductions in the progression of depigmentation (mean [90% CI]) %CFB) in the 50-mg group (+ 0.59 [–1.50, 2.68]; *P* = 0.0096; *N* = 136) and in the 30-mg group (–1.45 [–5.47, 2.57]; *P* = 0.0090; *N* = 33) at Week 24 vs. placebo (+ 5.68 [2.59, 8.76]; *N* = 56) (Fig. 3a, Table S5, Fig. S3). A progressive increase in depigmentation in the placebo group was observed in active lesions.

Within stable lesions, ritlecitinib treatment resulted in a statistically significant reduction in depigmentation (an increase in repigmentation) in the 50-mg group (–6.35 [–8.45, − 4.26]; *P* = 0.0016; *N* = 183) and in the 30-mg group (–7.98 [–12.95, − 3.01]; *P* = 0.0090; *N* = 28) at Week 24 compared with placebo (+ 0.51 [–2.89, 3.91]; *N* = 62) (Fig. 3b, Table S5, Fig. S3). Depigmentation did not change in the placebo group in stable lesions.

### Changes in biomarkers between active lesions and stable lesions following ritlecitinib

To examine the molecular effects of ritlecitinib on active and stable lesions in patients with active NSV, biopsied samples were analyzed by RNA-seq, qPCR, and IHC, and blood samples were analyzed by proteomics.

GSVA analysis revealed that both active and stable lesions displayed significantly (*P* < 0.01 and *P* < 0.1, respectively) decreased expression of Th1 markers (Fig. S4, markers were previously reported in patients with alopecia areata who were treated with ritlecitinib 50 mg [39]). At Week 24 compared with baseline, both active and stable lesions of patients in the 50-mg groups displayed a trend towards decreased expression of Th2 markers (Fig. S5, markers were previously reported in patients with alopecia areata [39]). RNA-seq analysis did not show statistically significant genes unique to active lesions or stable lesions in response to ritlecitinib treatment.

**Fig. 4:**
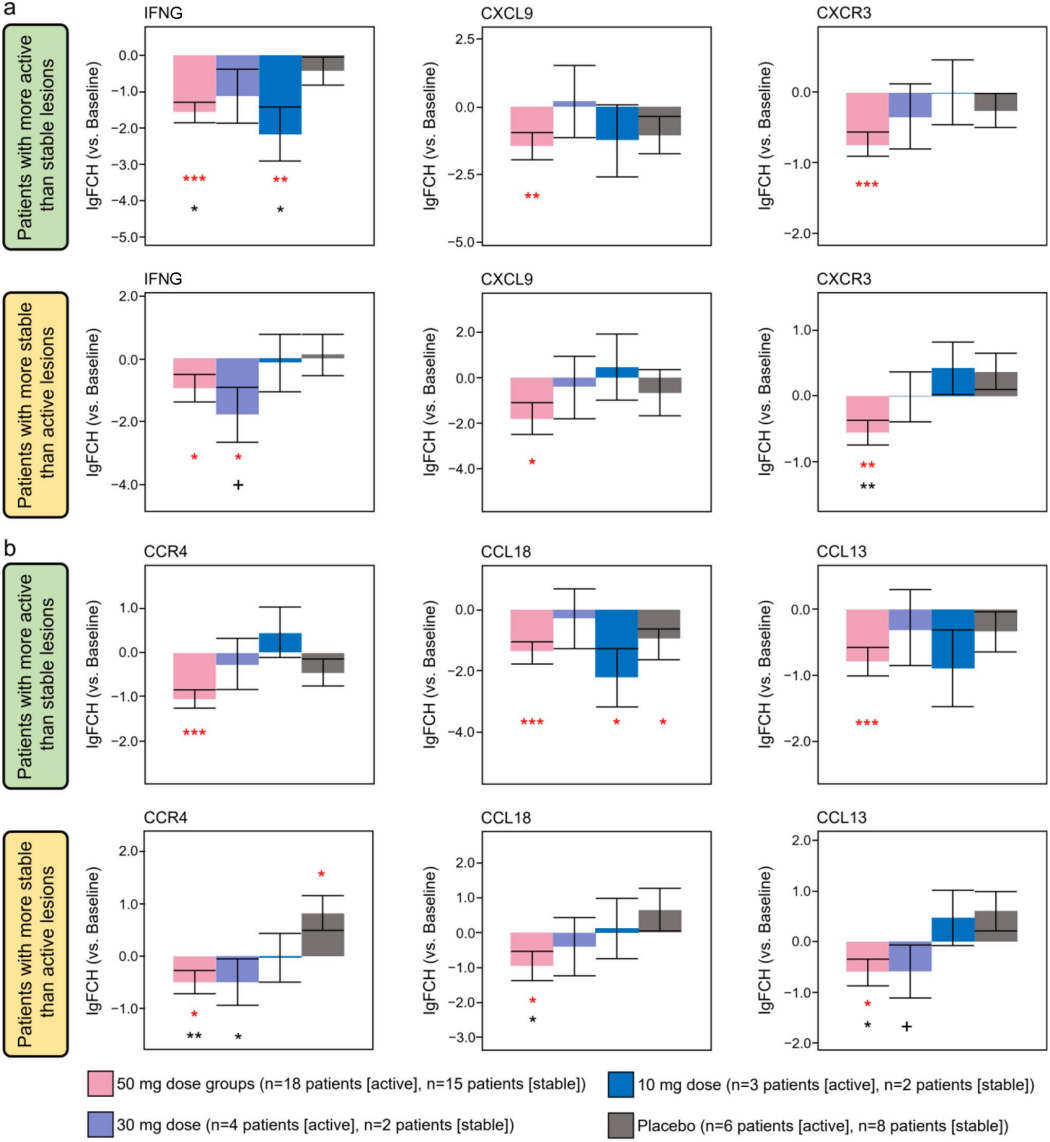
(**a**) Th1 markers and (**b**) Th2 markers by qPCR at Week 24 patients with more active than stable lesions and patients with more stable than active lesions (Cohort 2). lgFCH, log fold change; Th1, T helper Type 1; Th2, T helper Type 2. Red symbols: ^*^P < 0.05 vs. baseline; ** *P* < 0.01; *** *P* < 0.001. Black symbols: ^+^*P* < 0.1 vs. placebo; * *P* < 0.05 vs. placebo; ** *P* < 0.01

qPCR and TLDA were also performed for a large panel of immune and melanocyte markers to confirm RNA-seq results. When measured by qPCR, both active and stable lesions displayed significantly decreased expression of Th1 markers (IFNG, CXCL9, CXCR3; Fig. 4a, *P* < 0.05) and Th2 markers (CCR4, CCL18, and CCL13; Fig. 4b, *P* < 0.05) at Week 24 compared with baseline in patients in the 50-mg groups. However, qPCR and TLDA did not show statistically significant genes unique to active or stable lesions in response to ritlecitinib, although there was a trend towards increased levels of melanocyte markers (tyrosinase, TYRP1) in stable lesions vs. active lesions (data not shown, no statistical significance).

**Fig. 5:**
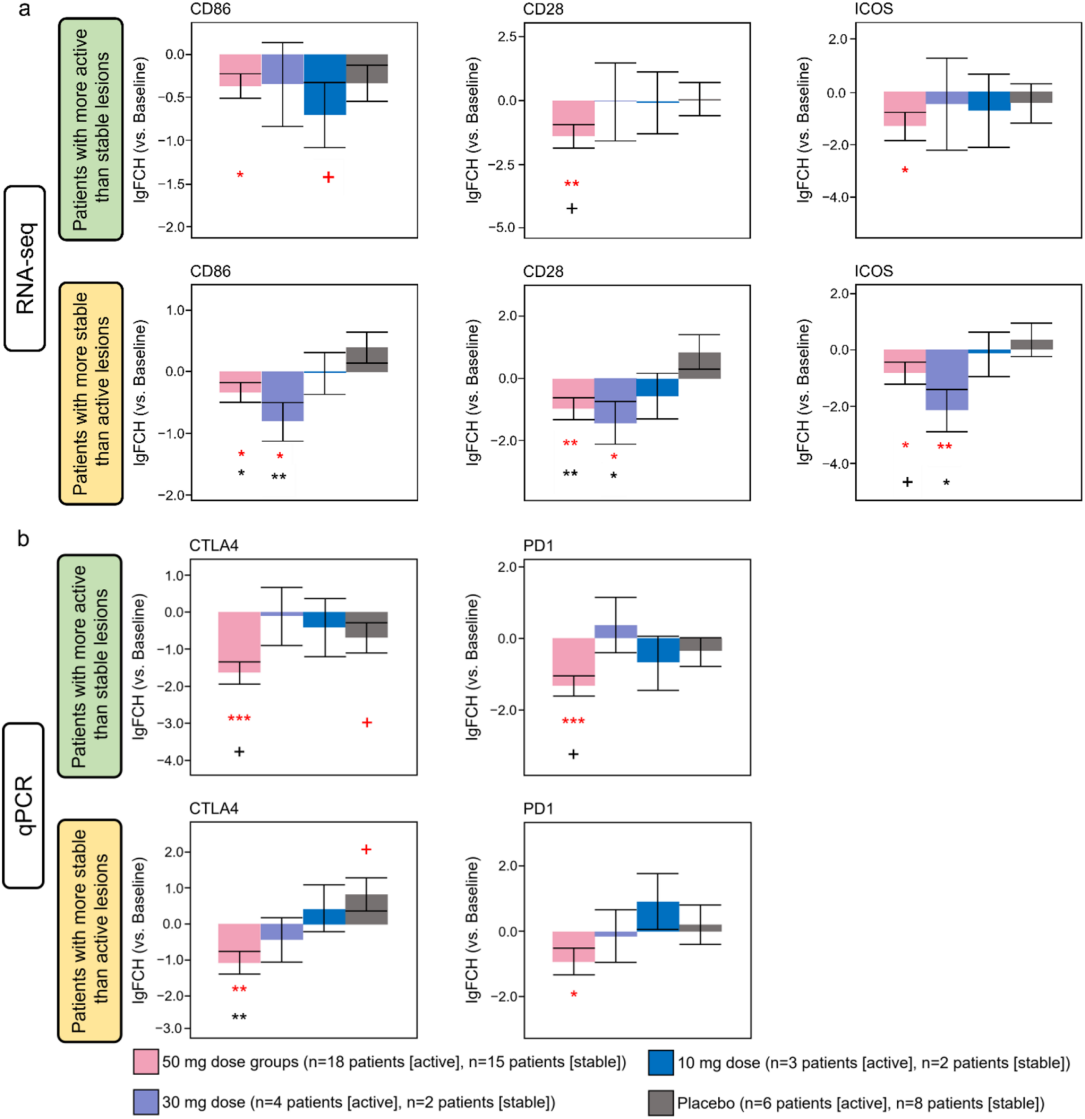
Co-stimulatory molecules by RNA-seq and qPCR at Week 24 in patients with more active than stable and patients with more stable than active lesions (Cohort 2). lgFCH, log fold change. Red symbols: ^+^*P* < 0.1 vs. baseline; *P < 0.05 vs. baseline; **P < 0.01 vs. baseline; ***P < 0.001 vs. baseline. Black symbols: ^+^*P* < 0.1 vs. placebo; *P < 0.05 vs. placebo; **P < 0.01 vs. placebo

We previously reported decreased expression levels of co-stimulatory molecules in response to ritlecitinib [32]. Here, we next investigated co-stimulatory molecules in lesions of patients with active NSV using RNA-seq and qPCR to examine any differences between active lesions and stable lesions. Both active lesions and stable lesions in the 50-mg groups displayed significantly decreased expression of co-stimulatory molecules and T-cell activation molecules such as CD86, CD28, inducible T-cell co-stimulator (ICOS), CTLA4, and PD-1 at Week 24 compared with baseline (Fig. 5, *P* < 0.05). A significant decrease in co-stimulatory molecules CD86, CD28, and ICOS in stable lesions was observed in the 30-mg group at Week 24 vs. baseline and vs. placebo (*P* < 0.05). This trend was not observed in active lesions.

At Week 24, stable lesions of patients in the ritlecitinib 50-mg group displayed a trend towards increased number of melanocytes in the epidermis compared with placebo when measured by IHC using melanocyte markers TYRP1 and Melan-A (*P* < 0.1, Fig. S6a). Active lesions also displayed a trend towards increase in number of melanocytes but to a small extent, as compared with stable lesions (no statistical significance between active and stable lesions at Week 24). Additionally, at Week 24 patients in the ritlecitinib 50-mg dose groups showed a significant reduction in T-cell infiltrates in both stable and active lesions (Fig. S6b, *P* < 0.05). Patients receiving placebo displayed no trend toward reduction in T-cell infiltrates.

**Fig. 6:**
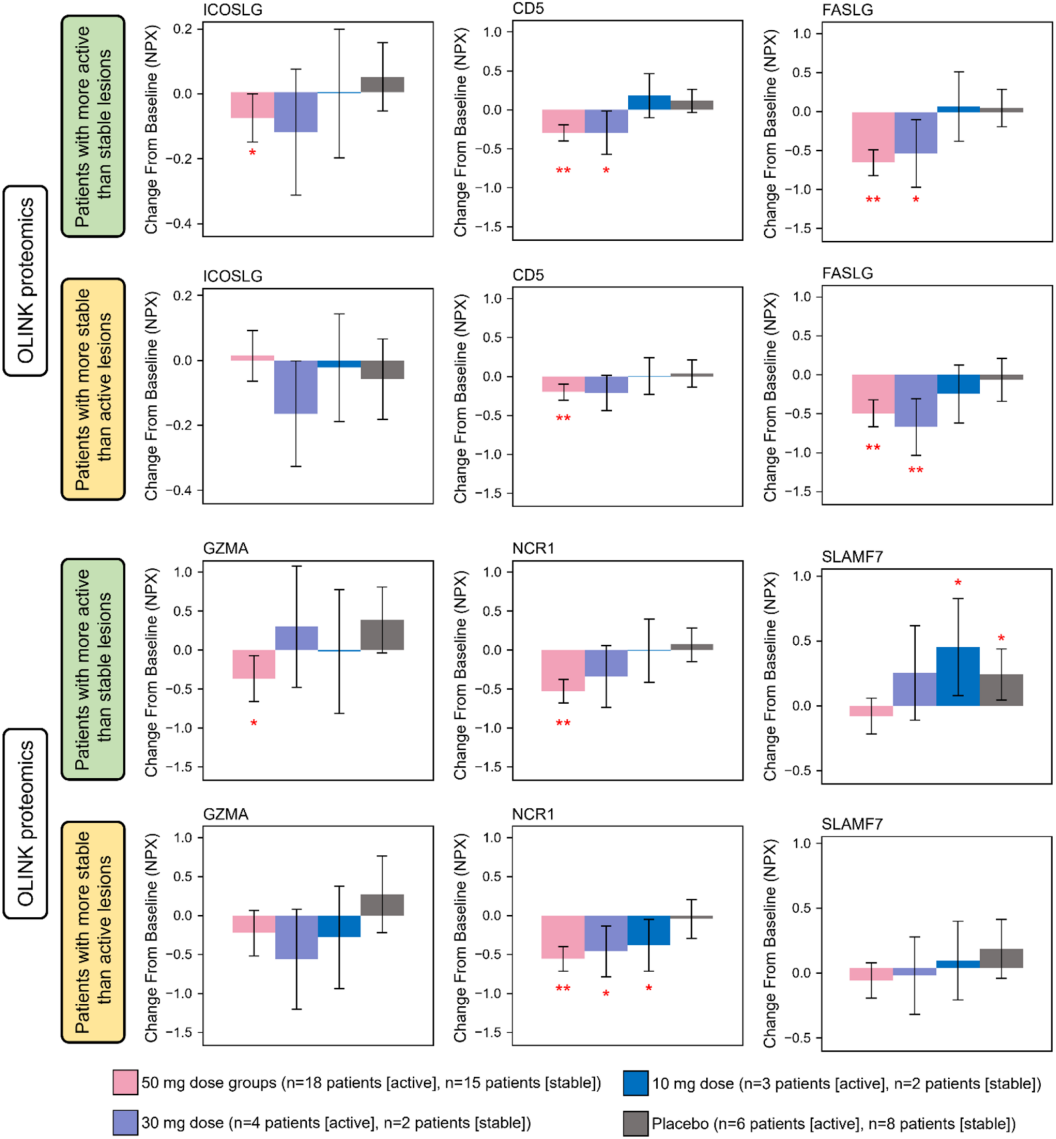
Change from baseline in serum levels of ICOSLG, NK cell activation markers, and inflammatory marker by proteomics at Week 24 in patients with more active than stable and patients with more stable than active lesions (Cohort 2). NPX, normalized protein expression. *P < 0.05 vs. baseline; **P < 0.01 vs. baseline

Finally, we investigated serum inflammatory/immune markers using proteomics to examine any differences between patients with more active than stable lesions and patients with more stable than active lesions in response to ritlecitinib. Compared with baseline, serum from patients with more active than stable lesions receiving 50-mg ritlecitinib showed a significant decrease from baseline in the ICOS marker ICOSLG at Week 24 (Fig. 6, *P* ≤ 0.05). All patients who received 50-mg or 30-mg ritlecitinib showed significant decreases from baseline in markers of NK cell activation (Fig. 6, *P* < 0.05). In patients with more active than stable lesions who received 10 mg or placebo, significant increases from baseline in levels of inflammatory marker SLAMF7 at Week 24 were observed (Fig. 6, *P* < 0.05).

## Discussion

Clinically active vitiligo lesions can be differentiated from stable lesions macroscopically based on the presence of confetti-like lesions, trichrome lesions, or Koebner phenomenon types 2a/b [37]. We hypothesized that many key inflammatory biomarkers contributing to the formation of active vitiligo lesions could be identified at the gene and protein levels, as compared with stable lesions, by investigating skin and serum samples from patients with active NSV. However, the differences between active lesions and stable lesions at baseline were minimal. Statistically significant differences were only observed in the protein levels of CD103 (through IHC) and the expression levels of *IFNG* and *CCL5* (through qPCR). Differences were only observed through RNA-seq analysis when the genes were compared between lesions vs. non-lesion (delta-comparison) but not by comparison of either lesions, non-lesions, or “lesions vs. non-lesions in patients with a more active than stable lesions” vs. “lesions vs. non-lesions in patients with a more stable than active lesions” (delta-delta comparison). Since most of the genes identified here have not previously been identified as a marker associated with melanogenesis or vitiligo pathogenesis, future studies are required to confirm these findings.

Gene expression-level analyses showed higher expression levels of *IFN-γ* and *CCL5* in active lesions than stable lesions. As IFN-γ plays a crucial role in vitiligo pathogenesis [40], it is not surprising that inflamed active lesions express higher levels of *IFN-γ*. There was also increased *CCL5* expression in the active lesions [41]. IHC analysis showed higher protein levels of CD103 (integrin αE) in the epidermis of active lesions compared with stable lesions. Future studies may elucidate the role of CD103 in active vitiligo lesions.

At baseline, serum from patients with more active than stable lesions showed significantly higher levels of CXCL9 and PD-L1 and a lower level of HO-1 than serum from patients with more stable than active lesions. Previously, increased CXCL9 serum levels were reported in patients with active lesions [14], as CXCL9 together with CXCL10 plays key roles in the activation and recruitment of CD8^+^/CXCR3^+^ T cells [42]; however, this is the first report showing increased PD-L1 and decreased HO-1 serum levels in patients with more active than stable lesions. Although the PD-1/PD-L1 pathway is well studied in melanoma, and it has been shown that anti-PD-1 and anti-PD-L1 therapy may result in depigmentation in patients with melanoma, this pathway has not been fully elucidated in vitiligo [43]. Since ritlecitinib resulted in decreased PD-1 expression in both active and stable lesions, downregulating the PD-1/PD-L1 pathway may have a therapeutic effect on patients with vitiligo. By contrast, HO-1 is an antioxidant that participates in the oxidative stress response [44] and is a functional modulator of Tregs in vitiligo [45]. The level of HO-1 in Tregs is decreased in patients with vitiligo and treatment of Tregs with Hemin, an agonist of HO-1, restored Treg function in vitro [45]. Enhancing serum HO-1 levels via an agonist may show clinical efficacy in patients with active vitiligo, although further investigations are required.

This was the first study focusing on the differential effects between active and stable lesions in patients with active vitiligo treated with a JAK3/TEC family kinase inhibitor on both clinical measures (depigmentation) and molecular signatures. In active lesions, placebo-treated patients showed progressive depigmentation at Week 24, whereas the progression of depigmentation was halted in those receiving 50-mg or 30-mg ritlecitinib, suggesting that ritlecitinib stabilizes vitiligo progression in active lesions. Repigmentation of active lesions may occur at a later stage than 24 weeks, suggesting that longer studies are needed to fully understand the effects of ritlecitinib on repigmentation of active lesions. By contrast, repigmentation of stable lesions was observed in patients receiving ritlecitinib for 24 weeks, whereas no improvement was observed with placebo. The degree of improvement with ritlecitinib compared with placebo was similar between active lesions and stable lesions. Thus, oral ritlecitinib treatment is beneficial in patients with both active and stable lesions, although longer treatment is required to fully observe the efficacy in active lesions.

Changes in melanocyte biomarkers from skin biopsies of active and stable lesions were consistent with the clinical findings. These results indicate that both types of lesions display CD3^+^ T-cell and CD8^+^ T-cell infiltrates, although active lesions trended towards greater numbers of infiltrates. Additionally, gene expression–based analyses showed that the response to ritlecitinib was similar between stable and active lesions in terms of decreased expression levels of Th1 markers, Th2 markers, and co-stimulatory molecules. As we did not observe many significant differences between active and stable lesions at baseline, the key factors contributing to the difference between active/progressive lesions and stable lesions may be limited, and overall inflammation may be similar between stable and active vitiligo lesions.

Serum from patients with more active than stable lesions showed reduced levels of ICOSLG, a co-stimulatory molecule, and SLAMF7, an inflammatory marker expressed on CD8^+^ T cells, B cells, and NK cells [46] in response to ritlecitinib; these reductions were not observed in patients with more stable than active lesions. These differences in activation and inflammatory molecules may contribute to the faster response of stable lesions than active lesions, although further investigations are necessary.

This study had some limitations. The treatment period was only 24 weeks, and the efficacy and molecular effects of longer-term therapy remain to be evaluated, particularly on active lesions that may require longer treatment to increase melanocyte markers and achieve repigmentation. Additionally, the number of patients for the biomarker portion of this study was limited. Lastly, patients were required to have ≥ 1 active lesion; therefore, patients who had only stable lesions were not evaluated.

Collectively, these data provide for the first time clinical and molecular evidence that systemic ritlecitinib stabilizes active lesions while promoting repigmentation of stable lesions in patients with vitiligo.

## Electronic supplementary material

Below is the link to the electronic supplementary material.

Supplementary Material 1

Supplementary Material 2

## Data Availability

Upon request, and subject to review, Pfizer will provide the data that support the findings of this study. Subject to certain criteria, conditions and exceptions, Pfizer may also provide access to the related individual de-identified participant data. See https://www.pfizer.com/science/clinical-trials/trial-data-and-results for more information. Biomarker-specific data are available from the corresponding author upon reasonable request.
