# Supplementary material for: Ritlecitinib, a JAK3/TEC family kinase inhibitor, stabilizes active lesions and repigments stable lesions in vitiligo
Source: Arch Dermatol Res. 2024 Jul 18;316(7):478. doi: 10.1007/s00403-024-03182-y (PMC11258076; doi:10.1007/s00403-024-03182-y)
Supplement: Supplementary file 1 — Supplementary Material 1 [file 403_2024_3182_MOESM1_ESM.docx]

**SUPPLEMENTARY INFORMATION**

**3Ritlecitinib, a JAK3/TEC family kinase inhibitor, stabilizes active lesions and repigments stable lesions in vitiligo**

Yuji Yamaguchi^1^, Elena Peeva^2^, Ester Del Duca^3^, Paola Facheris^3,4^, Jonathan Bar^3,5^, Ronald Shore^6^, Lori Ann Cox^2^, Abigail Sloan^7^, Diamant Thaçi^8^, Anand Ganesan^9^, George Han^10^, Khaled Ezzedine^11^, Zhan Ye^2^, Emma Guttman-Yassky^3^

*^1^Inflammation & Immunology Research Unit, Pfizer, Collegeville, PA, USA; ^2^Inflammation & Immunology Research Unit, Pfizer, Cambridge, MA, USA; ^3^Department of Dermatology, and Laboratory of Inflammatory Skin Diseases, Icahn School of Medicine, Mount Sinai, New York, NY, USA; ^4^IRCCS Humanitas Research Hospital, Milan, Italy; ^5^Faculty of Medicine, Tel-Aviv University, Tel-Aviv, Israel; ^6^Ronald N. Shore Dermatology, Rockville, MD, USA; ^7^Clinical Statistics, Pfizer, Cambridge, MA, USA; ^8^Institut fuer Entzuendungsmedizin, University of Luebeck, Luebeck, Germany; ^9^Department of Dermatology, University of California, Irvine, Irvine, CA, USA; ^10^Department of Dermatology, Zucker School of Medicine at Hofstra/Northwell, New Hyde Park, NY, USA; ^11^Department of Dermatology, Hôpital Henri Mondor, Créteil, France*

**Corresponding author:** Dr. Emma Guttman-Yassky

Email: [emma.guttman@moutsinai.org](mailto:emma.guttman@moutsinai.org)

**TABLES**

**Table S1** Antibodies used for immunohistochemistry

| Characteristic | Manufacturer | Clone | Isotype | Dilution |
| --- | --- | --- | --- | --- |
| CD3 | BD Biosciences | SK7 | IgG1 | 1:100 |
| CD8 | BD Biosciences | HIT8a | IgG1 | 1:100 |
| MELAN-A | Invitrogen | A103 | IgG1 | 1:100 |
| TYRP1 | Abcam | TA99 | IgG2a | 1:100 |

**Table S2** Baseline characteristics of Cohort 1 (Ezzedine et al., 2022)

|  | **Ritlecitinib** | | | | |  | |
| --- | --- | --- | --- | --- | --- | --- | --- |
|  | **200/50 mg** | **100/50 mg** | **50 mg** | **30 mg** | **10 mg** | **Placebo** | **Total** |
| No. of patients | 65 | 67 | 67 | 50 | 49 | 66 | 364 |
| Male, n (%) | 35 (53.8) | 36 (53.7) | 28 (41.8) | 22 (44.0) | 24 (49.0) | 26 (39.4) | 171 (47.0) |
| Age, mean (SD), y | 45.4 (12.2) | 44.2 (11.2) | 43.3 (10.4) | 44.7 (13.5) | 46.6 (10.0) | 46.1 (11.5) | 45.0 (11.5) |
| Race, n (%) |  |  |  |  |  |  |  |
| Asian | 15 (23.1) | 17 (25.4) | 17 (25.4) | 5 (10.0) | 11 (22.4) | 21 (31.8) | 86 (23.6) |
| Black | 3 (4.6) | 0 | 0 | 4 (8.0) | 1 (2.0) | 2 (3.0) | 10 (2.7) |
| Other | 0 | 1 (1.5) | 1 (1.5) | 1 (2.0) | 2 (4.1) | 1 (1.5) | 6 (1.7) |
| White | 44 (67.7) | 47 (70.1) | 45 (67.2) | 39 (78.0) | 33 (67.3) | 38 (57.6) | 246 (67.6) |
| Not reported | 3 (4.6) | 2 (3.0) | 4 (6.0) | 1 (2.0) | 2 (4.1) | 4 (6.1) | 16 (4.4) |

**Table S3** Baseline characteristics of biopsy sub-study (Cohort 2 and 7 additional patients who had similar numbers of stable lesions and active lesions at baseline) (Guttman-Yassky E. et al., 2023)

|  | **Ritlecitinib** | | | | |  | |
| --- | --- | --- | --- | --- | --- | --- | --- |
|  | **200/50 mg** | **100/50 mg** | **50 mg** | **30 mg** | **10 mg** | **Placebo** | **Total** |
| No. of patients | 13 | 12 | 11 | 8 | 6 | 15 | 65 |
| Male, n (%) | 5 (38) | 7 (58) | 6 (55) | 6 (75) | 3 (50) | 4 (27) | 31 (48) |
| Age, mean (SD), y | 44.5 (12.3) | 42.8 (8.8) | 40.9 (9.6) | 45.5 (13.0) | 47.3 (4.6) | 48.7 (9.3) | 44.9 (10.2) |
| Race, n (%) |  |  |  |  |  |  |  |
| Asian | 2 (15) | 1 (8.3) | 0 | 0 | 0 | 4 (27) | 7 (11) |
| Black | 1 (7.7) | 0 | 0 | 1 (12) | 0 | 0 | 2 (3.1) |
| Other | 0 | 1 (8.3) | 0 | 1 (12) | 0 | 0 | 2 (3.1) |
| White | 8 (62) | 9 (75) | 9 (82) | 5 (62) | 5 (83) | 8 (53) | 44 (68) |
| Not reported | 2 (15) | 1 (8.3) | 2 (18) | 1 (12) | 1 (17) | 3 (20) | 10 (15) |

**Table S4** Differentially expressed genes between non-lesional and lesional skin at baseline in Cohort 2

|  | Gene | **Adjusted *P*-Value in active lesions** | **Adjusted *P*-Value in stable lesions** | **Adjusted *P*-Value in All lesions** |
| --- | --- | --- | --- | --- |
| Downregulated in active lesions | *PKNOX2* | 4.36e-05 |  | 2.15e-09 |
|  | *KCNJ13* | 8.52e-05 |  | 4.19e-05 |
|  | *ARSI* | 2.08e-04 |  | 6.00e-07 |
|  | *SLITRK2* | 1.53e-03 |  | 3.64e-07 |
|  | *C1QL2* | 4.79e-03 |  | 3.32e-03 |
|  | *CDH3* | 6.95e-03 |  | 2.79e-05 |
|  | *CYSLTR2* | 9.59e-03 |  | 5.15e-04 |
|  | *UGT3A2* | 3.27e-02 |  | 5.54e-03 |
|  | *C1QL1* | 4.70e-02 |  | 7.59e-03 |
| Upregulated in active lesions | *PNN* | 1.38e-07 |  | 1.75e-13 |
|  | *STX16-NPEPL1* | 3.28e-06 |  | 2.81e-08 |
|  | *ITGA2B* | 4.91e-04 |  | 3.54e-04 |
|  | *TRIM74* | 2.16e-03 |  | 1.32e-02 |
|  | *ZMAT1* | 3.17e-03 |  | 4.74e-05 |
|  | *NEFH* | 5.09e-03 |  | 1.49e-02 |
|  | *ACSM2A* | 8.74e-03 |  | 1.80e-02 |
|  | *IGF2* | 9.57e-03 |  | 4.83e-03 |
|  | *CCDC110* | 1.57e-02 |  | 2.71e-03 |
|  | *VIP* | 2.12e-02 |  | 5.68e-03 |
|  | *INSM2* | 2.92e-02 |  | 2.76e-02 |
|  | *DCAF4L1* | 2.94e-02 |  | 3.32e-02 |
|  | *RBM14-RBM4* | 3.16e-02 |  | 9.97e-02 |
|  | *ADGB* | 3.37e-02 |  | 1.09e-02 |
|  | *IZUMO1* | 3.74e-02 |  | 1.14e-02 |
|  | *NEXN* | 4.20e-02 |  | 4.07e-03 |
| Downregulated in stable lesions | *KCNAB2* |  | 5.22e-11 | 4.45e-14 |
|  | *L1CAM^a^* |  | 9.00e-06 | 8.46e-09 |
|  | *GJB1^a^* |  | 1.03e-05 | 2.19e-06 |
|  | *MSC* |  | 2.52e-05 | 5.31e-07 |
|  | *TSPAN10^a^* |  | 6.46e-05 | 3.62e-06 |
|  | *LRRTM1* |  | 1.90e-04 | 1.10e-02 |
|  | *NRXN3* |  | 1.94e-04 | 5.37e-06 |
|  | *FOXD3^a^* |  | 3.17e-04 | 5.71e-05 |
|  | *GREB1^a^* |  | 5.37e-04 | 2.59e-05 |
|  | *NLGN1* |  | 5.98e-04 | 3.83e-05 |
|  | *PCDH11X* |  | 6.09e-04 | 5.33e-04 |
|  | *CAPN3^a^* |  | 7.04e-04 | 3.73e-04 |
|  | *CTNNA2* |  | 9.61e-04 | 1.58e-04 |
|  | *TMEM151A* |  | 1.03e-03 | 1.05e-04 |
|  | *KIAA1755* |  | 1.60e-03 | 1.71e-03 |
|  | *KCNA2* |  | 2.89e-03 | 1.33e-03 |
|  | *DRP2* |  | 3.25e-03 | 1.23e-03 |
|  | *CMTM5* |  | 3.61e-03 | 2.45e-04 |
|  | *C10orf90* |  | 3.93e-03 | 8.63e-03 |
|  | *CERS1* |  | 6.54e-03 | 9.61e-04 |
|  | *HIST1H1E* |  | 8.21e-03 | 1.11e-03 |
|  | *CHRM1^a^* |  | 1.08e-02 | 2.10e-03 |
|  | *BMPR1B* |  | 1.21e-02 | 1.03e-03 |
|  | *PPEF1* |  | 1.28e-02 | 7.36e-02 |
|  | *RASGEF1C^a^* |  | 1.43e-02 | 1.12e-03 |
|  | *SCRG1* |  | 1.98e-02 | 2.01e-02 |
|  | *CHST9* |  | 2.02e-02 | 2.44e-03 |
|  | *MPZ* |  | 2.08e-02 | 1.40e-02 |
|  | *S100A1* |  | 2.08e-02 | 6.21e-03 |
|  | *CEND1* |  | 2.69e-02 | 1.93e-02 |
|  | *ROPN1B* |  | 2.83e-02 | 7.24e-03 |
|  | *RAP1GAP^a^* |  | 2.89e-02 | 2.54e-03 |
|  | *UNC5C* |  | 3.03e-02 | 6.51e-02 |
|  | *CCDC184* |  | 3.59e-02 | 2.04e-02 |
|  | *C9orf50* |  | 3.59e-02 | 2.07e-02 |
|  | *SMIM1* |  | 3.60e-02 | 5.06e-02 |
|  | *SLC8A2^a^* |  | 3.94e-02 | 4.09e-02 |
|  | *TPBGL* |  | 4.36e-02 | 3.88e-02 |
|  | *MAGEL2^a^* |  | 4.64e-02 | 2.30e-02 |
| Upregulated in stable lesions | *KTN1* |  | 2.07e-09 | 1.08e-14 |
|  | *LARP7^a^* |  | 1.76e-07 | 1.91e-12 |
|  | *LRRCC1* |  | 1.67e-06 | 2.89e-10 |
|  | *CIR1* |  | 2.16e-05 | 1.65e-09 |
|  | *CCDC18* |  | 8.96e-05 | 6.10e-06 |
|  | *POSTN* |  | 1.75e-03 | 1.43e-05 |
|  | *HMMR^a^* |  | 8.80e-03 | 2.75e-03 |
|  | *KCNH6* |  | 1.76e-02 | 4.92e-02 |
|  | *NEFM* |  | 4.49e-02 | 7.77e-02 |
|  | *AKR1B10* |  | 7.43e-02 | 3.29e-02 |
|  | *CLDN22* |  | 7.99e-02 | 7.70e-02 |
|  | *MS4A14* |  | 9.95e-02 | 1.48e-02 |
| Downregulated in both active and stable lesions | *PMEL^a^* | 4.23e-15 | 2.87e-14 | 5.65e-24 |
|  | *DCT^a^* | 4.23e-15 | 6.41e-15 | 7.87e-24 |
|  | *SFTPC* | 4.23e-15 | 4.10e-13 | 1.66e-22 |
|  | *CA14^a^* | 8.17e-14 | 1.72e-13 | 6.46e-22 |
|  | *PCSK2* | 9.06e-14 | 1.54e-12 | 1.33e-21 |
|  | *TYRP1^a^* | 2.80e-13 | 4.75e-14 | 1.46e-21 |
|  | *LGI3^a^* | 2.80e-13 | 8.26e-13 | 3.31e-21 |
|  | *SLC24A5^a^* | 5.17e-14 | 1.72e-13 | 4.92e-21 |
|  | *CLEC12B^a^* | 1.94e-13 | 6.62e-12 | 2.18e-20 |
|  | *SOX1* | 4.27e-13 | 1.99e-10 | 7.54e-20 |
|  | *KIF1A^a^* | 1.28e-11 | 2.45e-12 | 1.45e-19 |
|  | *SLC45A2^a^* | 1.97e-11 | 6.93e-12 | 2.42e-19 |
|  | *FCRLA* | 5.73e-12 | 8.03e-11 | 6.66e-19 |
|  | *SLC6A17* | 1.11e-10 | 2.23e-12 | 1.00e-18 |
|  | *APBA2* | 1.40e-09 | 1.43e-11 | 2.81e-18 |
|  | *PLP1* | 1.19e-08 | 3.98e-12 | 3.98e-18 |
|  | *BCAN* | 2.39e-11 | 5.57e-12 | 7.00e-18 |
|  | *PLXNC1* | 1.16e-09 | 9.46e-11 | 7.90e-18 |
|  | *GAPDHS* | 8.73e-10 | 6.79e-11 | 1.15e-17 |
|  | *ASB11* | 6.95e-10 | 1.72e-10 | 1.37e-17 |
|  | *SLC1A4* | 1.16e-09 | 2.55e-10 | 1.37e-17 |
|  | *CRABP1* | 1.07e-08 | 3.53e-11 | 6.04e-17 |
|  | *GPR143* | 4.54e-10 | 1.15e-09 | 1.06e-16 |
|  | *TRPM1^a^* | 4.54e-10 | 9.46e-11 | 1.22e-16 |
|  | *OCA2^a^* | 9.48e-11 | 3.67e-08 | 1.34e-16 |
|  | *IRF4^a^* | 7.99e-09 | 7.95e-10 | 1.80e-16 |
|  | *MFSD12^a^* | 1.99e-08 | 4.16e-10 | 1.80e-16 |
|  | *KIAA1549L* | 4.11e-08 | 1.07e-09 | 1.04e-15 |
|  | *MLANA^a^* | 1.41e-09 | 1.75e-10 | 2.31e-15 |
|  | *LZTS1* | 5.23e-08 | 3.39e-09 | 3.11e-15 |
|  | *TRIM67* | 5.26e-07 | 2.12e-10 | 3.32e-15 |
|  | *GPM6B* | 2.03e-08 | 7.03e-08 | 1.07e-14 |
|  | *DIPK1C* | 3.92e-07 | 2.32e-09 | 1.43e-14 |
|  | *TYR^a^* | 1.72e-09 | 2.21e-09 | 2.08e-14 |
|  | *TRIM63^a^* | 2.46e-06 | 8.06e-10 | 2.52e-14 |
|  | *GMPR* | 4.93e-06 | 9.38e-10 | 3.72e-14 |
|  | *SLC6A15* | 6.60e-06 | 2.82e-10 | 4.11e-14 |
|  | *EPHA5* | 2.05e-07 | 3.36e-08 | 4.18e-14 |
|  | *ARNT2* | 2.64e-06 | 3.29e-09 | 6.46e-14 |
|  | *CTXND1* | 1.24e-07 | 6.56e-08 | 7.43e-14 |
|  | *PLPPR4* | 3.34e-04 | 1.09e-10 | 3.23e-13 |
|  | *BIRC7* | 2.31e-05 | 3.56e-09 | 3.77e-13 |
|  | *MMP17* | 1.68e-05 | 7.78e-08 | 2.83e-12 |
|  | *SEP3* | 3.39e-06 | 5.72e-07 | 3.67e-12 |
|  | *C1QL4* | 1.07e-08 | 2.27e-04 | 4.54e-12 |
|  | *MPP4* | 3.53e-07 | 7.10e-05 | 3.01e-11 |
|  | *VEPH1* | 9.84e-05 | 1.97e-07 | 3.30e-11 |
|  | *KIT^a^* | 6.75e-04 | 6.77e-08 | 7.01e-11 |
|  | *PAX3^a^* | 1.86e-04 | 3.70e-07 | 1.03e-10 |
|  | *EDNRB^a^* | 7.15e-05 | 1.81e-05 | 7.25e-10 |
|  | *OR7A5* | 6.75e-04 | 1.71e-06 | 1.11e-09 |
|  | *SOX10^a^* | 1.75e-03 | 7.24e-07 | 1.30e-09 |
|  | *BEST1* | 2.12e-03 | 5.03e-07 | 1.32e-09 |
|  | *ALX1* | 9.05e-05 | 3.53e-05 | 1.81e-09 |
|  | *GSG1L* | 1.20e-04 | 2.94e-05 | 2.00e-09 |
|  | *TUBB4A* | 7.69e-03 | 4.32e-07 | 4.61e-09 |
|  | *MCHR1^a^* | 7.20e-06 | 1.39e-03 | 5.08e-09 |
|  | *LINGO1^a^* | 1.26e-03 | 2.18e-05 | 1.43e-08 |
|  | *CRYM* | 3.22e-03 | 1.76e-05 | 3.22e-08 |
|  | *TUBB3^a^* | 4.30e-03 | 1.41e-05 | 3.90e-08 |
|  | *WDR63* | 2.73e-03 | 3.72e-05 | 4.67e-08 |
|  | *PKHD1* | 2.46e-03 | 1.05e-04 | 9.95e-08 |
|  | *SLC5A10^a^* | 5.67e-04 | 8.28e-04 | 1.36e-07 |
|  | *OR7C1* | 3.86e-03 | 2.89e-04 | 4.00e-07 |
|  | *LTK* | 6.75e-04 | 2.94e-03 | 5.27e-07 |
|  | *KCNN2* | 3.29e-04 | 2.10e-02 | 2.19e-06 |
|  | *GALNTL6* | 3.13e-03 | 4.28e-03 | 3.42e-06 |
|  | *DPP6* | 2.30e-02 | 4.12e-04 | 3.60e-06 |
|  | *COL24A1* | 4.85e-03 | 2.89e-03 | 3.62e-06 |
|  | *MDGA2* | 3.19e-03 | 7.49e-03 | 6.17e-06 |
|  | *NAT8L* | 6.87e-02 | 1.90e-04 | 6.50e-06 |
|  | *ZNF280B* | 4.40e-02 | 4.86e-04 | 8.91e-06 |
|  | *IGSF11* | 4.15e-02 | 6.97e-04 | 1.05e-05 |
|  | *GRIK1* | 3.84e-03 | 2.97e-02 | 3.14e-05 |
|  | *SYT6* | 3.68e-02 | 3.39e-03 | 4.08e-05 |
|  | *SLC35F1* | 2.75e-02 | 8.32e-03 | 5.80e-05 |
|  | *TFAP2B^a^* | 3.21e-02 | 9.01e-03 | 7.66e-05 |
|  | *KCNH8* | 1.80e-02 | 3.25e-02 | 1.46e-04 |
|  | *ST8SIA6* | 5.34e-02 | 3.49e-02 | 5.05e-04 |
|  | *FRZB* | 9.16e-02 | 2.30e-02 | 6.21e-04 |
|  | *AMER2* | 6.10e-02 | 4.51e-02 | 7.65e-04 |
|  | *NUDT10* | 5.84e-02 | 9.05e-02 | 1.56e-03 |
| Upregulated in both active and stable lesions | *PPIG* | 4.54e-10 | 3.34e-11 | 1.42e-18 |
|  | *NSRP1* | 1.72e-09 | 1.08e-09 | 6.38e-17 |
|  | *UPF3B* | 1.84e-07 | 6.88e-08 | 4.88e-14 |
|  | *HMGN5* | 1.96e-06 | 7.73e-06 | 2.08e-11 |
|  | *DNAJC2* | 1.21e-05 | 1.74e-06 | 2.25e-11 |
|  | *CEP290* | 6.74e-06 | 4.37e-06 | 3.05e-11 |
|  | *MNS1* | 2.33e-03 | 7.72e-07 | 1.90e-09 |
|  | *EIF5B* | 9.73e-05 | 5.35e-05 | 2.14e-09 |
|  | *XAF1^a^* | 6.80e-04 | 6.97e-04 | 1.31e-07 |
|  | *CFAP58* | 6.94e-03 | 9.81e-04 | 2.00e-06 |
|  | *SRRM5* | 6.72e-03 | 4.39e-03 | 7.47e-06 |
|  | *NME8* | 9.59e-03 | 4.96e-02 | 1.30e-04 |

^a^ Gene associated with melanocyte development/regulation or vitiligo (based on PubMed search).

**Table S5** Mean %CFB of clinical depigmentation in lesions at Week 24 in Cohort 1

|  | **Active Lesions** | | | | **Stable Lesions** | | | |
| --- | --- | --- | --- | --- | --- | --- | --- | --- |
| **Summary statistics** | **Ritlecitinib 200/50 mg, 100/50 mg,  50 mg** | **Ritlecitinib 30 mg** | **Ritlecitinib 10 mg** | **Placebo** | **Ritlecitinib 200/50 mg, 100/50 mg,  50 mg** | **Ritlecitinib 30 mg** | **Ritlecitinib 10 mg** | **Placebo** |
| No. of lesions | 136 | 33 | 32 | 56 | 183 | 28 | 44 | 62 |
| Estimated least squares mean (SE) | 0.59 (1.27) | –1.45 (2.44) | 1.59 (2.48) | 5.68 (1.87) | –6.35 (1.27) | –7.98 (3.01) | –3.29 (2.41) | 0.51 (2.06) |
| 90% CI | (–1.50, 2.68) | (–5.47, 2.57) | (–2.51, 5.68) | (2.59, 8.76) | (–8.45, –4.26) | (–12.95, –3.01) | (–7.26, 0.67) | (–2.89, 3.91) |
| Difference vs placebo |  |  |  |  |  |  |  |  |
| Least squares mean difference (SE) | –5.08 (2.16) | –7.13 (2.99) | –4.09 (3.02) | - | –6.87 (2.31) | –8.50 (3.57) | –3.81 (3.10) | – |
| 90% CI | (–8.65, –1.52) | (–12.07, –2.18) | (–9.07, 0.89) | - | (–10.67, –3.06) | (–14.39, –2.6) | (–8.92, 1.31) | – |
| One-sided unadjusted *P*-value | 0.0096 | 0.0090 | 0.0882 | - | 0.0016 | 0.0090 | 0.1103 | – |

CFB, change from baseline.

**FIGURES**

**Fig. S1** (a) Quantile-quantile plot showing the empirically observed quantiles of gene differentiated between lesional and non-lesional skin in active lesion (red) and stable lesion (blue) skin samples (y-axis) at baseline in Cohort 2 as a function of quantiles expected from a normal distribution with the same mean and variance as the empirical distribution(x-axis). (b) Quantile-quantile plot showing the empirically observed quantiles of gene tested between the differences of lesion vs non-lesional skin samples in patients with more active than stable lesions versus the differences of lesion vs non-lesional skin samples in patients with more stable than active lesions (y-axis, Cohort 2) at baseline as a function of quantiles expected from a normal distribution with the same mean and variance as the empirical distribution (x-axis).

**
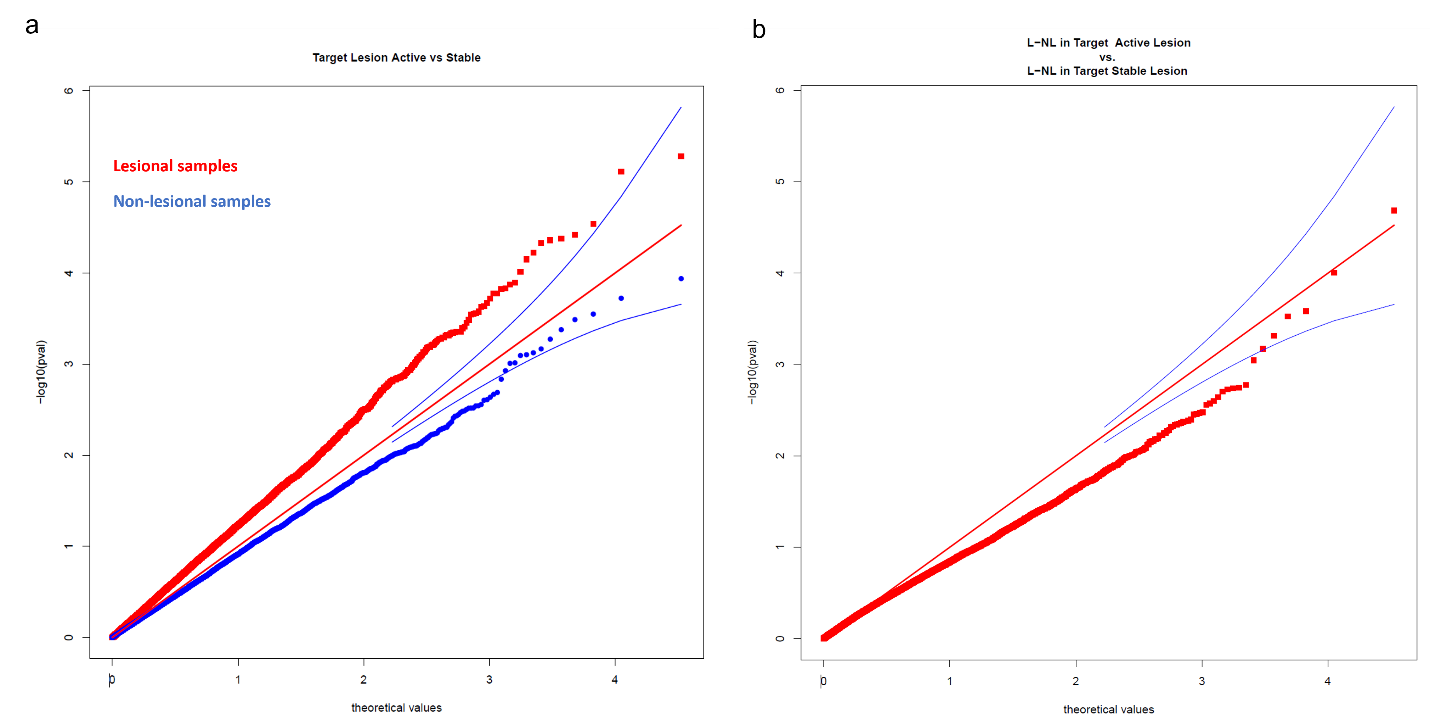
**

**Fig. S2** Differential expression of biomarkers at baseline in biopsies from active and stable lesions of Cohort 2, as measured by qPCR and/or TLDA, and IHC


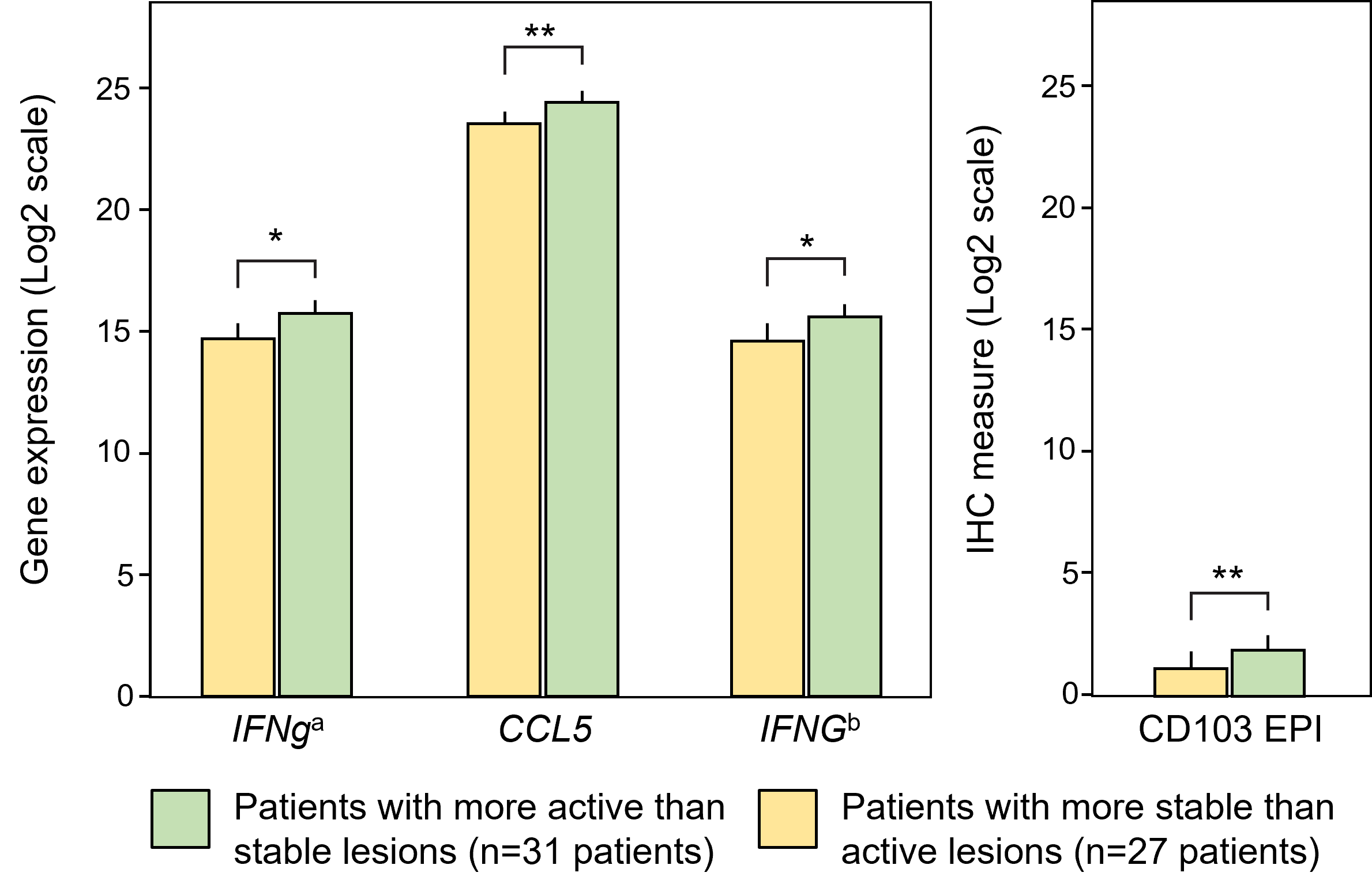


At baseline in Cohort 2, active lesions showed higher expression of *IFNG* and *CCL5,* and protein levels of CD103 than stable lesions.

^a^Measured by qPCR

^b^Measured by TLDA

EPI, epidermis; IHC, immunohistochemistry; qPCR, quantitative real-time qPCR; TLDA, TaqMan low-density array.

**Fig. S3** Representative images of active and stable vitiligo lesions at baseline and Week 24 in a patient treated with ritlecitinib 200/50 mg (belonging to Cohorts 1 and 2)

**
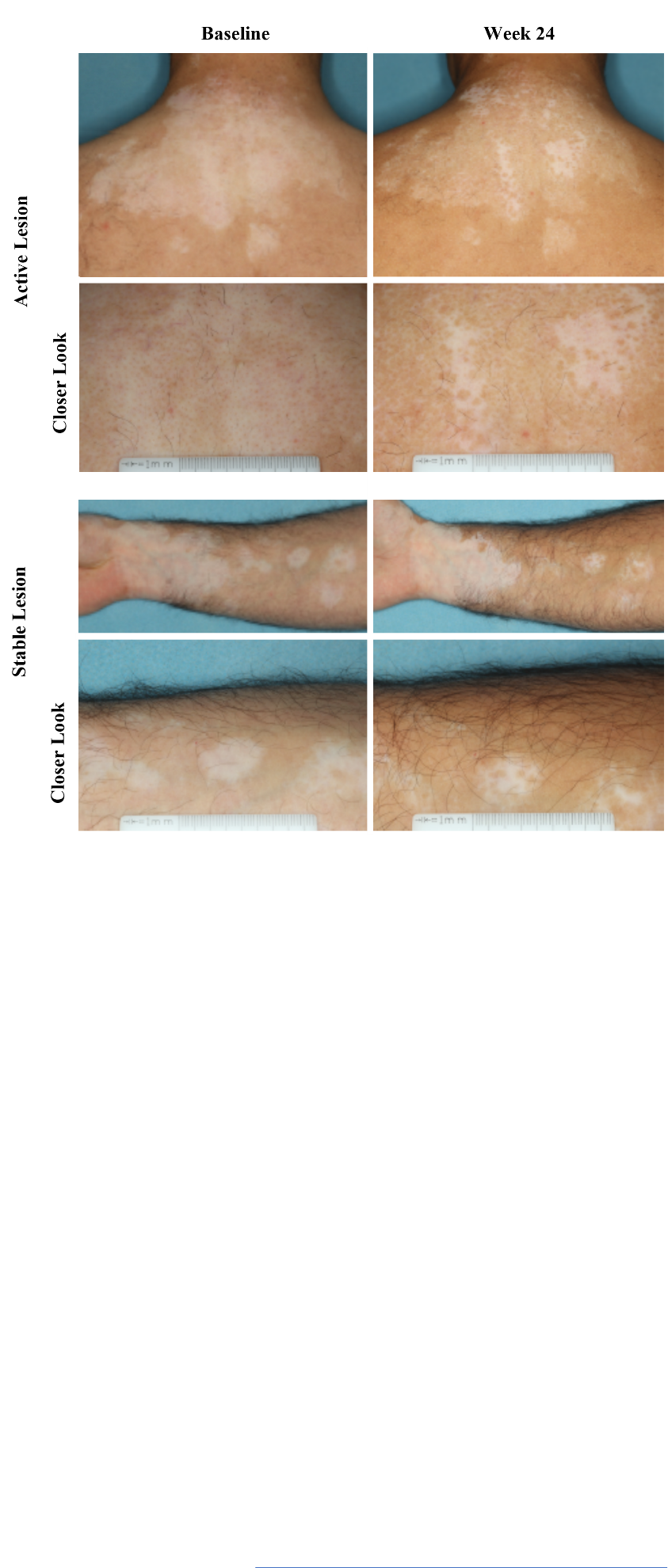
**

At baseline, this subject showed a greater number of active lesions than stable lesions (categorized as a patient with more active than stable lesions) with a total VASI of 16.1 at baseline. Back lesions were active with trichrome lesions and confetti-like lesions (with trunk-VASI of 5.4), while right forearm lesions were stable with demarcated lesions (with upper extremities-VASI of 2.0) at baseline. After 24 weeks of ritlecitinib treatment, this subject showed an improvement with total VASI of 7.1, trunk-VASI of 2.0, and upper extremities-VASI of 1.5. Patient was a representative of Cohort 2 and was categorized as having more active than stable lesions and a few stable lesions.

VASI, Vitiligo area scoring index.

**Fig. S4** Th1 markers by RNA-seq at Week 24 vs baseline in Cohort 2

**
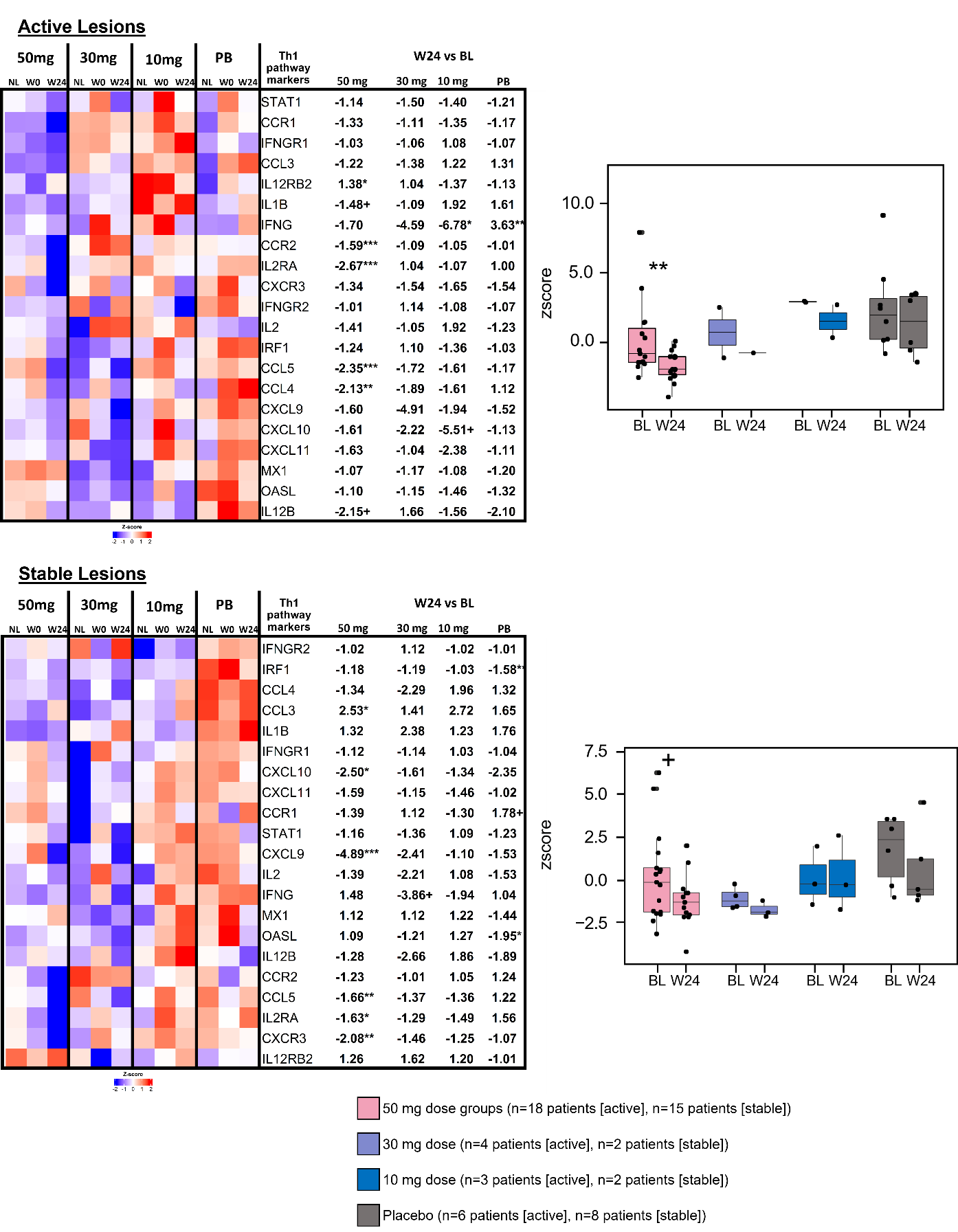
**

BL, baseline; PB, placebo; Th1, T helper Type 1; W24, Week 24.

^+^ *P*<0.1; ** *P*<0.01.

**Fig. S5** Th2 markers by RNA-seq at Week 24 vs baseline in Cohort 2

**
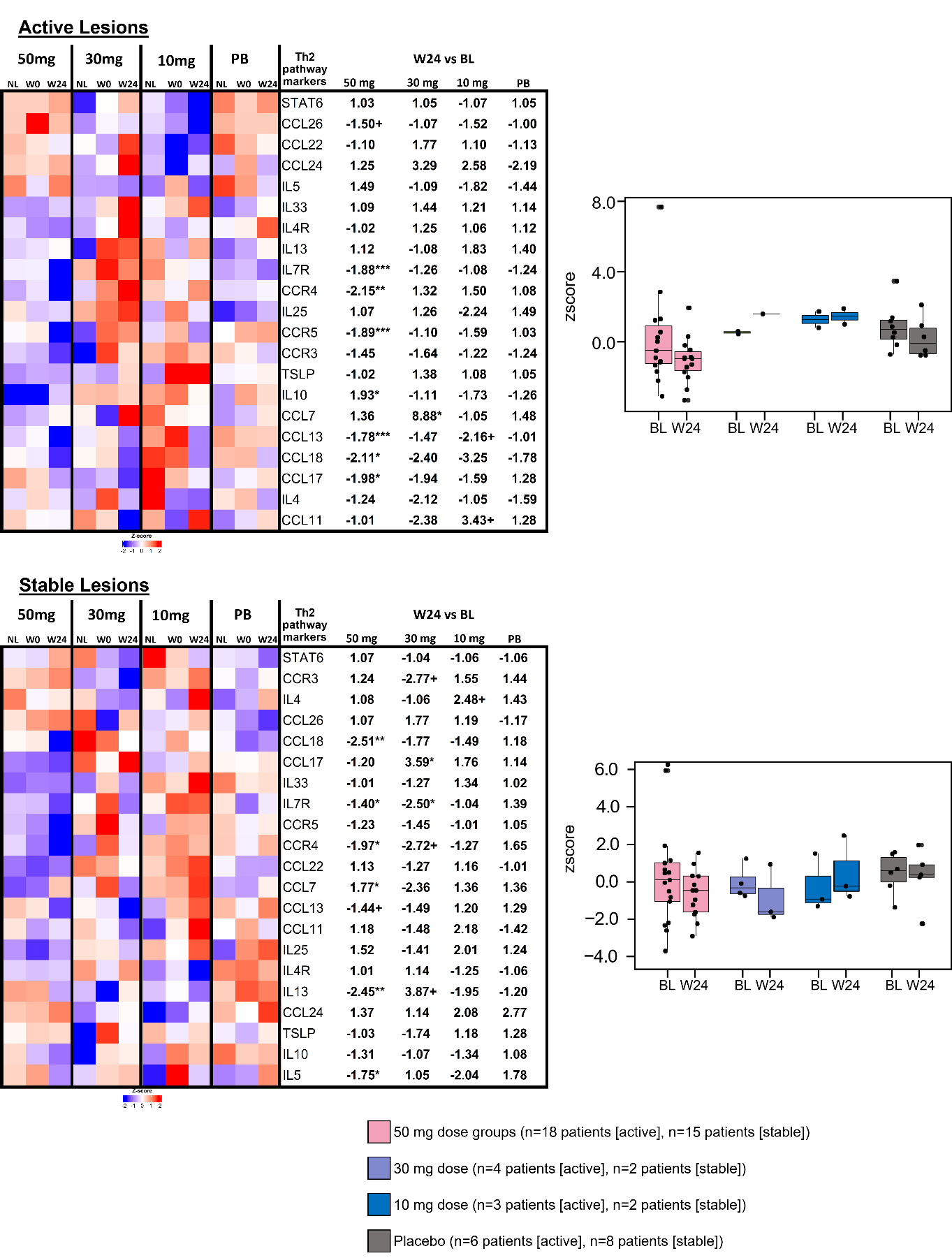
**

BL, baseline; PB, placebo; Th2, T helper Type 2; W24, Week 24.

**Fig. S6** (a) Melanocyte marker protein levels and (b) CD3 and CD8 protein levels by IHC at Week 24 in active lesions and stable lesions from a representative patient of Cohort 2

**
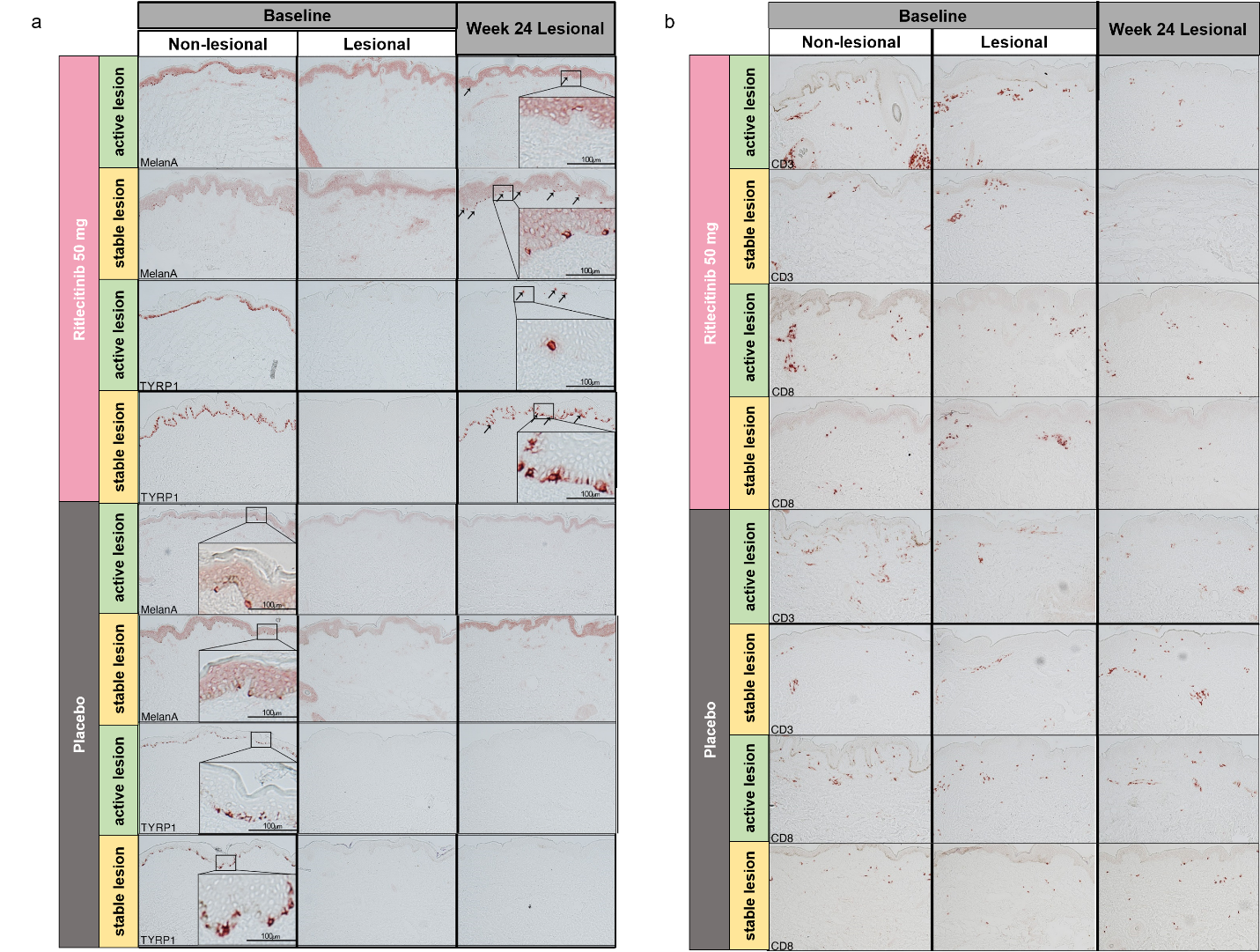
**

(a) A trend towards an increase in expression of melanocyte markers was observed in patients receiving 50 mg ritlecitinib daily. (b) In patients receiving 50 mg ritlecitinib daily, both active and stable lesions showed a reduction in infiltrates. Placebo baseline images correspond to those in Fig. 2.
