# Supplementary material for: Ritlecitinib, a JAK3/TEC family kinase inhibitor, stabilizes active lesions and repigments stable lesions in vitiligo
Source: Arch Dermatol Res. 2024 Jul 18;316(7):478. doi: 10.1007/s00403-024-03182-y (PMC11258076; doi:10.1007/s00403-024-03182-y)
Supplement: Supplementary file 2 — Supplementary Material 2 [file 403_2024_3182_MOESM2_ESM.pptx]

## Slide 1
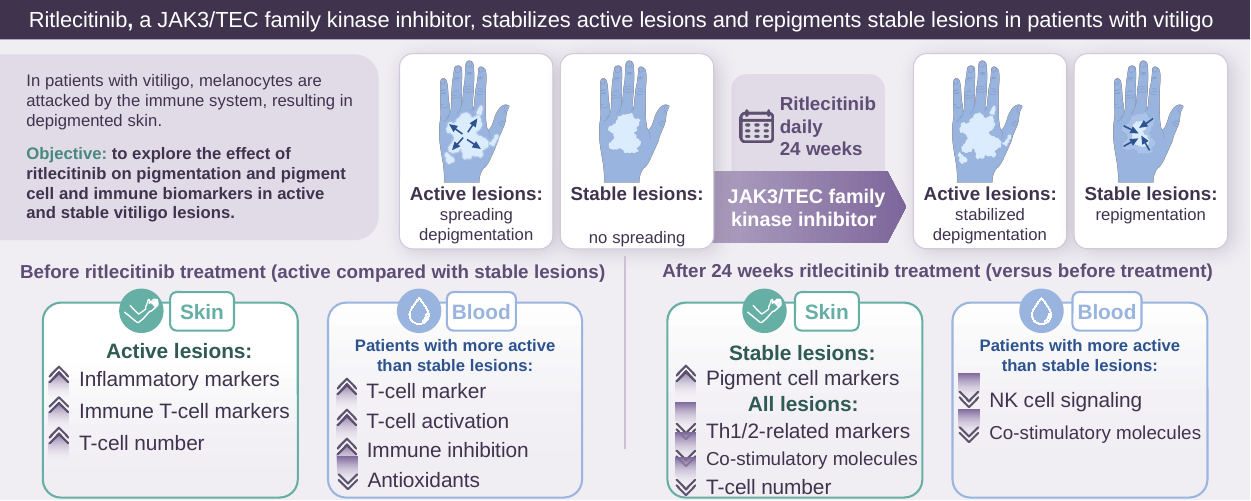

Ritlecitinib, a JAK3/TEC family kinase inhibitor, stabilizes active lesions and repigments stable lesions in patients with vitiligo
In patients with vitiligo, melanocytes are attacked by the immune system, resulting in depigmented skin.
Objective: to explore the effect of ritlecitinib on pigmentation and pigment cell and immune biomarkers in active and stable vitiligo lesions.
Ritlecitinib
daily
24 weeks
JAK3/TEC family kinase inhibitor
Active lesions:
spreading depigmentation
Stable lesions:
no spreading
Active lesions:
stabilized depigmentation
Stable lesions:
repigmentation
After 24 weeks ritlecitinib treatment (versus before treatment)
Before ritlecitinib treatment (active compared with stable lesions)
Skin
Blood
Skin
Blood
Patients with more active than stable lesions:
Patients with more active than stable lesions:
Active lesions:
Stable lesions:
Pigment cell markers
Inflammatory markers
NK cell signaling
T-cell marker
All lesions:
Immune T-cell markers
Th1/2-related markers
T-cell activation
Co-stimulatory molecules
T-cell number
Co-stimulatory molecules
Immune inhibition
T-cell number
Antioxidants
